# Intermittent preventive treatment in pregnancy with sulfadoxine–pyrimethamine and parasite resistance: cross-sectional surveys from antenatal care visit and delivery in rural Ghana

**DOI:** 10.1186/s12936-022-04124-7

**Published:** 2022-03-26

**Authors:** Atikatou Mama, Charity Ahiabor, Bernard Tornyigah, Naa Adjeley Frempong, Kwadwo A. Kusi, Bright Adu, David Courtin, Sandrine Houzé, Philippe Deloron, Michael F. Ofori, Abraham K. Anang, Frédéric Ariey, Nicaise Tuikue Ndam

**Affiliations:** 1 Université de Paris, MERIT, IRD, 75006 Paris, France; 2 Department of Parasitology, Noguchi Memorial Institute for Medical Research, College of Health Sciences, University of Ghana, Legon, Ghana; 3 INSERM 1016, Service de Parasitologie-Mycologie Hôpital Cochin, Université de Paris, 75014 Paris, France; 4 Department of Immunology, Noguchi Memorial Institute for Medical Research, College of Health Sciences, University of Ghana, Legon, Ghana; 5 Department of Science Laboratory Technology, Faculty of Applied Sciences, Accra Technical University, P O Box GP 561, Accra, Ghana; 6 Centre National de Référence du Paludisme, Hôpital Bichat-Claude Bernard, APHP, 75018 Paris, France

**Keywords:** Malaria, Pregnancy, *Plasmodium falciparum*, Ghana, IPTp-SP, SP resistance

## Abstract

**Background:**

Despite decades of prevention efforts, the burden of malaria in pregnancy (MiP) remains a great public health concern. Sulfadoxine-pyrimethamine (SP), used as intermittent preventive treatment in pregnancy (IPTp-SP) is an important component of the malaria prevention strategy implemented in Africa. However, IPTp-SP is under constant threat from parasite resistance, thus requires regular evaluation to inform decision-making bodies.

**Methods:**

In two malaria endemic communities in the Volta region (Adidome and Battor), a cross-sectional hospital-based study was conducted in pregnant women recruited at their first antenatal care (ANC) visit and at delivery. Basic clinical and demographic information were documented and their antenatal records were reviewed to confirm IPTp-SP adherence. Peripheral and placental blood were assayed for the presence of *Plasmodium falciparum* parasites by quantitative polymerase chain reaction (qPCR). One hundred and twenty (120) positive samples were genotyped for mutations associated with SP resistance.

**Results:**

At first ANC visit, *P. falciparum* prevalence was 28.8% in Adidome and 18.2% in Battor. At delivery, this decreased to 14.2% and 8.2%, respectively. At delivery, 66.2% of the women had taken at least the recommended 3 or more doses of IPTp-SP and there was no difference between the two communities. Taking at least 3 IPTp-SP doses was associated with an average birth weight increase of more than 360 g at both study sites compared to women who did not take treatment (p = 0.003). The *Pfdhfr/Pfdhps* quintuple mutant **IRN**I-**A/FG**KAA was the most prevalent (46.7%) haplotype found and the nonsynonymous *Pfdhps* mutation at codon A581G was higher at delivery among post-SP treatment isolates (40.6%) compared to those of first ANC (10.22%). There was also an increase in the A581G mutation in isolates from women who took 3 or more IPTp-SP.

**Conclusions:**

This study confirms a positive impact following the implementation of the new IPTp-SP policy in Ghana in increasing the birth weight of newborns. However, the selection pressure exerted by the recommended 3 or more doses of IPTp-SP results in the emergence of parasites carrying the non-synonymous mutation on codon A581G. This constant selective pressure calls into question the time remaining for the clinical utility of IPTp-SP treatment during pregnancy in Africa.

## Background

In sub-Saharan Africa where malaria is still endemic, pregnant women commonly suffer from malaria in pregnancy (MiP) resulting in low birth weight (LBW), maternal anaemia, and increased risk of infant mortality during the first years of life. The pathophysiology of MiP is associated with the ability of *Plasmodium falciparum*, the species accounting for more than 90% of the world’s malaria mortality, to sequester in the intervillous spaces of the placenta through interaction with the parasite ligand VAR2CSA [1–5]. To reduce the burden of MiP on pregnant women in sub-Saharan Africa, the World Health Organization (WHO) has recommended a set of measures including the preventive treatment with sulfadoxine-pyrimethamine (SP) of all pregnant women from the second trimester of pregnancy [6–9]. Although the adaptation and use of intermittent preventive treatment of malaria in pregnancy (IPTp) has been shown to be effective in averting LBW across different SP-resistant settings [10, 11], this treatment has often fallen short of the WHO's expectations. Several studies showed that the two-dose regimen initially recommended was inadequate in protecting neonates from the deleterious effects of MiP during the third trimester [12–14]. This together with a beneficial dose-dependent effect of IPTp-SP on birth weight and the SP alternatives with similar advantages on birth weight caused the WHO to revise its policy and recommended a monthly uptake of IPTp-SP from the second trimester of pregnancy [12]. However, concern is growing as regards the effect of increased SP uptake on the emergence of drug-resistant malaria parasites. This has a particular resonance in sub-Saharan Africa, where there is already a noticeable increase and spread of resistance to SP [15] and to date, no other alternative drug with the same benefits as SP has been identified [16].

Indeed, a recent study mediation analysis of three trials comparing the overall malaria and non-malaria effects of IPTp-SP versus IPTp with dihydroartesunate-piperaquine (DP) showed that IPTp-DP was superior to IPTp-SP when considering malaria-specific endpoints, but IPTp-SP had better efficacy in improving birth weight [17]. Another study made in a low transmission area (The Gambia) also demonstrated that risk of LBW declined as the number of IPTp-doses increased and appeared lowest among women who received three doses [18].

Like most sub-Saharan countries, Ghana adopted and intensified the implementation of the revised WHO policy in 2013 [3, 19], with pregnant women taking a higher number of IPTp-SP doses starting from the second trimester of pregnancy. Thus, requiring regular evaluation to preserve the intended benefit of the policy change. In Ghana, a study carried out two years after the revised policy implementation by Quakyi and colleagues in urban and peri-urban areas showed a correlation between increased birth weight and increased uptake of IPTp-SP doses [5]. Another study in rural Northern Ghana demonstrated an association between increased IPTp-SP dosage (≥ 3) and a better birth outcome [8]. However, the majority of these investigations were carried out in urban communities where malaria infection is low and resources are abundant. In the present study, pregnant women were recruted in two malaria endemic rural and semi-urban areas in the Volta region of Ghana in order to assess IPTp-SP coverage, SP resistance, and its consequences on health of both mothers and their newborn infants.

## Methods

### Study sites

The study was conducted between November 2016 and March 2019 in two distinct malaria endemic communities of the Volta region of Ghana: Battor and Adidome. Battor is a semi-urban community and the administrative capital of the North Tongu District and situated 6° 4′ 0″ North, 0° 25′ 0″ East. The primary health care service in the district is mainly provided by the Battor Catholic Hospital with the support of local Community-based Health Planning and Services (CHIPS) compounds. Adidome is a rural community located 50 km East of Battor and is the administrative capital for the Central Tongu District. Primary health care is provided by the district hospital located in Adidome and also supported by a number of CHIPS compounds. Malaria transmission in these areas is perennial with two transmission peaks during rainy seasons in April-July and September–November. The entomological inoculation rate (EIR) for the Volta region was approximately 65 (95% CI: 0–143) infectious bites per person per year in 2008 [9] and the main vector is *Anopheles gambiae*.

### Study design and sample collection

The study was designed as hospital-based cross-sectional surveys to recruit pregnant women visiting Battor and Adidome hospital for their first prenatal visit (ANC) and for delivery. Standardized questionnaires and checklists were used to collect demographic and clinical data both at first ANC and delivery. At first ANC, only HIV-negative pregnant women who had not received any prophylaxis against malaria were recruited into the study after their consent were obtained. Peripheral blood was collected into an EDTA tube, as well as blood spots on filter paper before administration of IPTp-SP. Haemoglobin (Hb) levels were measured at the hospital laboratory, and results recorded in participants’ ANC booklets and ANC records books. The participants ANC booklets are pregnancy information booklets given to each pregnant women in order to facilitate the pregnancy follow-up. The ANC records are hospital’s files with records about all pregnant women coming at the hospital.

At delivery, the number of IPTp-SP dose uptake, parity status and complementary clinical data such as the number of ANC visits, any type of complication experienced during pregnancy, type of delivery, Hb level and birth weight were obtained from participants’ ANC booklets. Peripheral and placental blood from each participant was also collected into separate EDTA vacutainer tubes, blood spots were made and the mother’s peripheral blood Hb level measured at the hospital’s laboratory.

For all participants, parasite detection was performed at the hospital either by RDT when available or microscopy. All blood samples were centrifuged and each blood component (plasma and red blood cell pellets) stored either at − 20 °C or − 80 °C for further analysis.

Women recruited during ANC were included in the analysis if they had essential information such as age, parity status, maternal haemoglobin levels, as well as malaria qPCR results on the peripheral blood at the time of inclusion. At delivery, pregnant women were included in the analysis if, in addition to the above-mentioned criteria, there were records of the number of antenatal IPTp-SP doses administered, as well as malaria qPCR results for both peripheral and placental blood at the time of delivery.

### DNA extraction and parasite detection by PCR

DNA was extracted from the blood spots on filter paper using the Chelex method [20]. The presence of *P. falciparum* DNA in the extracted DNA was examined in duplicate by qPCR amplifying 18S rRNA, as previously described [21]. Samples without amplicons (no cycle thresholds detected) were considered negative. Purified parasite DNA from the 3D7 strain was used as a positive control, while a negative control with no DNA template was run in parallel.

### Genotyping of *Pfdhfr* and* Pfdhps*

The *Pfdhfr* and *Pfdhps* genes of *P. falciparum* were amplified as previously described [22]. The PCR products were purified and sequenced using the Sanger method (GATC, Cologne, Germany). All generated sequences were analysed with the Chromas software [23], then aligned using the Muscle software [24] and compared to the 3D7 *P. falciparum* reference genome.

### Statistical analysis

At delivery, the main variable of interest was IPTp-SP uptake, and it was grouped into 3 categories; women who did not receive any IPTp-SP dose, women who received 1 or 2 doses, and women who received ≥ 3 doses of IPTp-SP. Parasitological outcomes were compared between groups by univariate analysis and logistic or linear regression models. Changes in maternal haemoglobin levels and birthweight were assessed using linear regression models and stratified by IPTp-SP dosing. The adjustment factors were gravidity (primigravidae or multigravidae), infection status (positive or negative by PCR) anaemia status at time of sampling and SP uptake from the ANC booklet. Student’s t test and chi squared test were used to compare participant's baseline characteristics and to determine the association between the *pfdhps* A581G mutation and the number of doses of SP taken where appropriate.

P-values of less than 0.05 were considered as statistically significant. Stata version 13 was used for data analysis and GraphPad version 5.01 for graphical depiction of data.

## Results

### Study population characteristics

A total of 932 pregnant women were recruited at the first ANC and 617 women at delivery. At the end, 692 women out of 932 (366 from Adidome and 326 from Battor) were included in the analysis for ANC versus 499 (284 in Adidome and 215 in Battor) for women recruited at delivery. The characteristics of the participants were similar between the two study sites, except for age (*p* = 0.003), gestational age (*p* < 0.0001), and Hb level (*p* = 0.004) at ANC (Table 1). Pregnant women recruited at first ANC at Adidome were generally younger and had a lower gestational age at the time of their first antenatal care visits (Age = 25.81 ± 6.39; Gestational age = 13.93 ± 7.05) compared to those recruited at Battor (Age = 27.44 ± 6.01; Gestational age = 16.53 ± 8.20). Pregnant women from Battor had higher haemoglobin levels (11.36 ± 1.15) compared to those from Adidome (10.61 ± 1.44).

**Table 1:** General characteristics of the study population

Characteristics	1st ANC visit			p-value	Delivery			p-value
	Overall (n = 692)	Adidome (n = 366)	Battor (n = 326)		Overall (n = 499)	Adidome (n = 284)	Battor (n = 215)	
Age (Mean ± SD)	26.59 ± 5.14	25.81 ± 6.39	27.44 ± 6.01	**	26.98 ± 4.76	26.88 ± 6.07	27.26 ± 5.31	
Gravidity/Parity (n (%))								
Primigravidae	204 (29.48%)	114 (31.06%)	90 (27.69%)		117 (23.44%)	65 (23.13%)	52 (26.40%)	
Multigravidae	488 (70.52%)	253 (68.94%)	235 (72.31%)		361 (72.34%)	216 (76.87%)	145 (73.60%)	
Gestational age in weeks (Mean ± SD)	15.16 ± 5.86	13.93 ± 7.05	16.53 ± 8.20	***	38.84 ± 1.41	38.75 ± 1.78	38.98 ± 1.74	
Bed net possession (n (%))								
Yes	644 (93.06%)	338 (92.10%)	306 (94.74%)		454 (90.98%)	257 (94.83%)	197 (95.63%)	
No	46 (6.65%)	29 (7.90%)	17 (5.26%)		23 (4.61%)	14 (5.17%)	9 (4.37%)	
Bed net usage (n(%))								
Always	466 (67.34%)	238 (64.85%)	228 (70.59%)		267 (53.51%)	162 (60.90%)	105 (50.97%)	
Sometimes	177 (25.58%)	104 (28.34%)	73.0(22.6%)		169 (33.87%)	89 (33.46%)	80 (38.83%)	
Seldom	47 (6.79)	25 (6.81%)	22 (6.81%)		17 (3.41%)	5 (1.88%)	12 (5.83%)	
HB^x^ (Mean ± SD)	10.73 ± 1.13	10.61 ± 1.44	11.36 ± 1.15	**	10.77 ± 0.93	10.79 ± 1.22	10.71 ± 1.87	
Anaemia status (n (%))								
Severe anaemic (Hb < 8.0 g/dL)	14 (2.23%)	14 (4.6%)	0 (0%)		16 (3.21%)	8 (2.85%)	8 (5.88%)	
Anaemic (11.0 < Hb ≥ 8.0 g/dL)	168 (24.28%)	154 (50.66%)	14 (25%)		209 (41.88%)	135 (48.04%)	74 (54.41%)	
Birth Outcome (Mean ± SD)					3.11 ± 0.4	3.14 ± 0.52	3.05 ± 0.58	
Low birth weight < 2500 g					38 (7.62%)	21 (7.45%)	17 (9.82%)	

^*^p ≤ 0.05; **p < 0.01; ***p < 0.001

### *Plasmodium falciparum* prevalence

Of the women who attended their first ANC, only 77.3% (297/384) at Adidome and 81.9% (267/326) at Battor had microscopy data, because the results of some blood smears were considered uncertain due to poor quality and therefore excluded. For the women retained, *P. falciparum* prevalence detected by microscopy at Adidome and Battor was 12.5% (37/297) and 6.4% (17/267), respectively. When assessed by qPCR, the prevalence increased to 28.4% (104/366) and 18.1% (59/326) in Adidome and Battor, respectively.

At delivery, *P. falciparum* prevalence by microscopy was 0.7% (2/284) and 0% (0/215), respectively, and was 14.1% (40/284) and 7.9% (17/215) at Adidome and Battor when detected by qPCR. *P. falciparum* prevalence in placental samples determined by qPCR at delivery was 16.5% (47/284) at Adidome against 3.3% (7/215) at Battor (Table 2).

**Table 2:** *Plasmodium falciparum* prevalence at both study sites and at 1st ANC and Delivery

Characteristics	1st ANC			Delivery		
	Overall (n = 692)	Adidome (n = 366)	Battor (n = 326)	Overall (n = 499)	Adidome (n = 284)	Battor (n = 215)
Peripheral infection*						
By microscopy	52(9.22%)	37(12.42%)	15(5.64%)	2(0.4%)	2(3.77%)	**0**
By qPCR	163(23.55%)	104(28.4%)	59(18.1%)	57(11.42%)	40(14.1%)	17(7.9%)
Parasite density^**#**^						
By qPCR	3086 ± 5886	5308 ± 85,068	693 ± 6332	7320 ± 14,328	11,566 ± 66,809	4669 ± 34,805
Placental infection						
By qPCR				54(10.82%)	47(16.5%)	7(3.3%)

^*^n (%); ^#^Mean ± SD

### IPTp-SP coverage

Since IPTp-SP was given under direct observation therapy (DOT), information on uptake by pregnant women at delivery was obtained from their antenatal booklets as a source of IPTp-SP uptake. Among the 499 pregnant women recruited at delivery, 94.8% (473/499) had data on IPTp-SP uptake. Among these, 97% (459/473) took at least one dose of IPTp, 66.2% (313/473) took 3 or more IPTp-SP doses, while 31% (146/473) took one or two doses. Fourteen pregnant women reported no IPTp-SP uptake due to non-attendance at an ANC.

**Fig.1:**
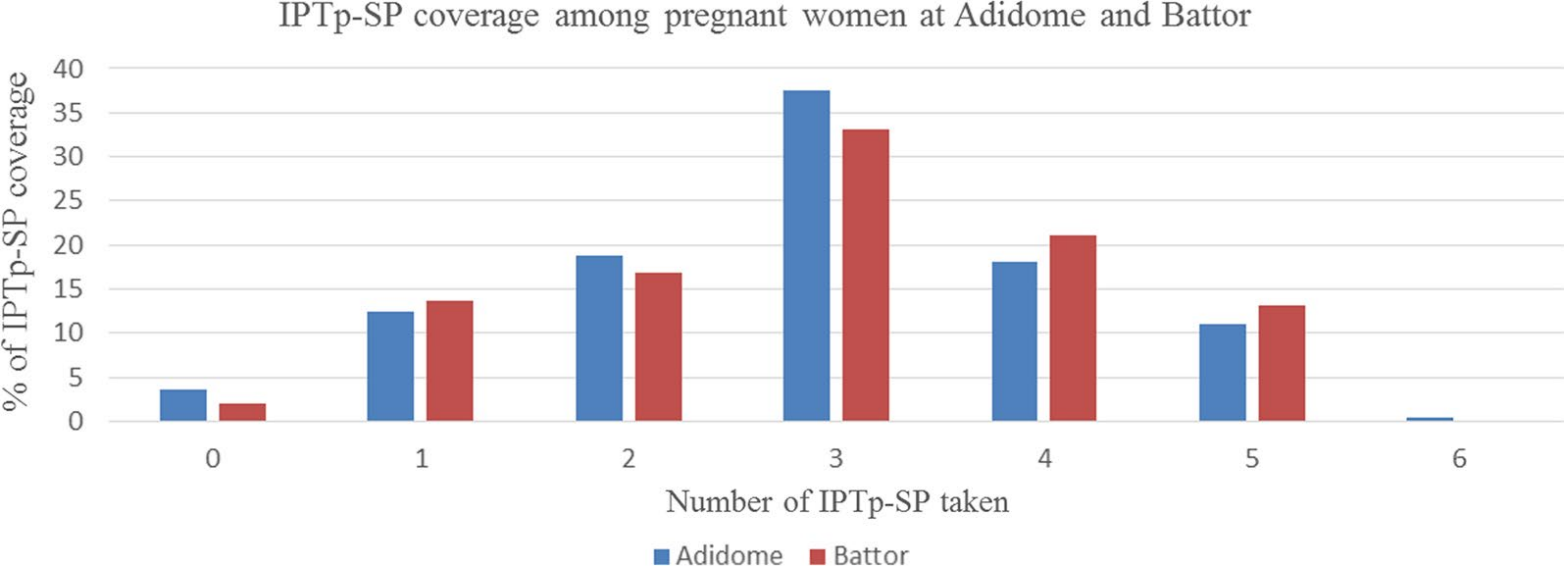
IPT_P_-SP coverage among pregnant women at Adidome and Battor. 0dose = Women who did not take SP during pregnancy Dose <  = 2 pregnant women who took 1 or 2 doses as recommended by the old regimen. Dose > 2 pregnant women who took at least 3 doses as recommended by the new regimen

When the data were stratified by study site, at Adidome 283 of the 284 women recruited had data on IPTp-SP uptake. One hundred and eighty-five women (65.4%) received 3 or more IPTp-SP doses, 18.7% (53/283) received two IPTp-SP doses and 12.4% (35/283) received one IPTP-SP dose. Ten women reported no IPTp-SP uptake due to non-attendance at ANC visit. At Battor, 88.4% (190/215) of pregnant women recruited at delivery had information on IPTp-SP uptake. Out of this number, 67.4% (128/190) received 3 or more IPTp-SP doses, 16.8% (32/190) received two IPTp-SP doses and 13.7% (26/190) received only one IPTp-SP dose. Four women reported no IPTp-SP due to non-attendance at ANC (Fig. 1).

### Impact of IPTp-SP uptake on birth weight

Overall multivariate regression analysis showed that uptake of 3 or more IPTp-SP doses increased the average birthweight by 360 g compared to babies born to mothers who did not take treatment (p = 0.03). Although an increase in birthweight of 250 g was also observed among infants born to women who took only 1 or 2 IPTp-SP doses, this was not statistically significant (p = 0.14). As expected, children born to primigravid mothers weighed on average 250 g less than those born to multigravidae (p < 0.0001) (Table 3).

**Table 3:** Relationship between birth weight and SP uptake by multivariate regression

	Coefficients (g)	95% CI	p-value
No SP uptake	Reference		
Overall effect			
1 or 2 IPTp-SP uptake^a^	250	[− 0.08; 0.57]	0.137
≥ 3 IPTp-SP uptake^b^	360	[0.04; 0.68]	0.028
Multigravidae	Reference		
Primigravidae	− 250	[− 0.36; − 0.13]	< 1e−04

^a^ SP uptake of less than 3 doses (1 or 2 SP uptake) reflecting the old IPTp-SP policy recommendation

^b^ SP uptake of 3 or more doses reflecting the proxy of the 2012 recommendation

### Prevalence of *Pfdhfr* and *Pfdhps* mutations

A total of 120 *P. falciparum* isolates were sequenced, of which, 73.3% (88/120) were from pregnant women recruited at ANC (58% from Adidome and 42. % from Battor) and 26.7% (32/120) from pregnant women recruited at Delivery (31.3% from Battor and 68.7% from Adidome). Of the 120 isolates sequenced, 93.3% (112/120) were successfully amplified, sequenced and genotyped for *Pfdhfr* and 87.5% (105/120) for *Pfdhps*.

A high prevalence of nonsynonymous mutations of three codons of *Pfdhfr* and two codons of *Pfdhps* was detected among the parasite isolates. Regarding *Pfdhfr*, the prevalence of a mutant allele at codon 108 (S108N, 98.2%, 110/112) was predominant; followed by that of codon 59 (C59R, 97.3%, 109/112) and 51 (N51I, 89.3%, 100/112). However, only one mutation was observed at codon 164 (I164L, 0.9%, 1/112). Similarly, a high prevalence of a mutation at codon 437 (A437G, 97.1% (102/105) of the *Pfdhps* gene was observed followed by that of 436 (S436A/F, 72.4%, 76/105). Additionally, mutations were observed respectively at codons 581 (A581G, 21%, 22/105) and 613 (A613S/T, 4.8%, 5/105). However, no mutation was detected at codon 540 (Table 4). The analysis was then focused on the nonsynonymous mutation A581G and showed that mutant alleles (A581G) were preferentially found at delivery following IPTp-SP treatment (40.6%), compared to inclusion (10.22%) (Ratio of first ANC/delivery 1:3.9; p = 0,001) (Table 5).

**Table 4:** Prevalence of *Pfdhfr, Pfdhps*, among pregnant women in study isolates

Gene	SNP^*^	Wild type, n (%)	Mutation, n (%)
*Pfdhfr*^*a*^ (n = 112)	N51I	12 (10.71%)	100 (89.29%)
	C59R	3 (2.68%)	109 (97.32%)
	S108N	2 (1.79%)	110 (98.21%)
	I164L	111 (99.11%)	1 (0.89%)
*Pfdhps*^*b*^ (n = 105)	S436A/F	29 (27.62%)	76 (72.38%)
	A437G	3 (2.86%)	102 (97.14%)
	K540E	105 (100%)	0
	A581G	82 (78.10%)	22 (20.90%)
	A613S/T	100 (95.24%)	5 (4.76%)

^a^ *Pfdhfr*, dihydrofolate reductase; ^b^ *Pfdhps*, dihydropteroate synthase

**Table 5:** A581G mutation at first ANC visit and at delivery

	No. of positive isolates (%)		p-value
Mutated codons at Dhps			
581	First ANC (n = 88)	Delivery (n = 32)	0.001
+	9 (10.22%)	13(40.6%)	

+ : presence of A581G mutation

When the *Pfdhfr/Pfdhps* haplotypes were combined for the 105 parasites for whom sequence data was obtained; 16 haplotypes were observed (Table 6). Isolates with quintuple mutations including triple *Pfdhfr* **IRN**I and double *Pfhps* **A/FG**KAA was the most common combined trait seen at a rate of 46.7% (49/105), followed by the quadruple combined trait (**IRN**I-S**G**KAA) at 16.2% (17/105). The occurrence of other combined genotypes was generally low and comprised between 1 and 8%.

**Table 6:** Prevalence of combined *pfdhfr*/*pfdhps* haplotypes in study isolates

Gene	category	Haplotype	n (%)
*pfdhfr*/*pfdhps* (n = 105)	wild type	NCSI-SAKAA	0
	double	IC**N**I_S**G**KAA	1 (0.95%)
	triple	N**RN**I-S**G**KAA	2(1.9%)
		IC**N**I-S**G**K**G**A	1 (0.95%)
	quadruple	**IRN**I-S**G**KAA	17 (16.19%)
		**IRN**I-**A/**FAKAA	3 (2.90%)
		**IRN**I-A**/F**AKAA	1 (0.95%)
		N**RN**I-**A/FG**KAA	8 (7.6%)
	quintuple	**IRN**I-**A/FG**KAA	49 (46.7%)
		**IRN**I_S**G**K**G**A	9 (8.57%)
		N**RN**I**_A/FG**K**G**A	1 (0.95%)
		**IR**SI_**A/FG**K**G**A	1 (0.95%)
	sixtuple	N**RN**I-A**/FG**K**GS/**T	1 (0.95%)
		**IRN**I-**A/FG**KA**S/**T	2 (1.90%)
		**IRN**I_**A/FG**K**G**A	6 (5.71%)
		**IRNL_**S**G**K**G**A	1 (0.95%)
	septuple	**IRN**I-**A/FG**K**GS/**T	2 (1.90%)

## Discussion

The SP drug, currently used as intermittent preventive treatment during pregnancy (IPTp-SP), remains a fundamental component in the strategy to protect pregnant women from malaria and its adverse effects on pregnancy outcome. Yet, its adaptation and utilization had been unexpectedly sub-optimal, while several studies showed its inability to provide protection from malaria, especially in the third trimester [25]. Combined, this prompted a policy review to increase its utilization and coverage throughout the pregnancy period, and since revision of this policy by the WHO, many countries including Ghana are working for its successful implementation. The initial and few evaluation studies indicate that the new treatment regimen is associated with a real clinical benefit on the outcome of pregnancy [5, 8]. However, such studies need to be repeated on a regular basis to monitor the preservation of this added value, as it could over time, lead to other suspected consequences on parasite resistance that could mitigate clinical benefit. The policy of increasing treatment doses certainly applies an increased drug-selection pressure on parasites and therefore, it must be regularly assessed to inform decision-makers.

Here, two different sets of pregnant women living in rural areas on the banks of the Volta river in Ghana were enrolled: those visiting health centres for their first ANC and the second set coming to deliver the babies at health centres. The design of the present study makes it possible to capture the characteristics of women appearing at their first ANC and to determine their usage of IPTp-SP, as part of the strategy for malaria prevention during pregnancy in the delivery cohort. The first observation is early attendance of ANC, women arriving on average between 14 and 16th week of pregnancy. This early attendance to an ANC is a determining factor for the successful implementation of the new IPTp-SP policy. Indeed, it is essential that women arrive early at an ANC to offer more time to receive a maximum of treatment doses over a longer period of pregnancy [26]. This observation in rural areas is particularly important, because it’s similar to that made in urban areas in a previous study by Quakyi et al. [5] and clearly indicates that sensitization of women in Ghana for their early participation in ANCs has been relayed across the country. This is in particular due to the National Malaria Control Programme’s (NMCP) efforts in promoting awareness combined with improved education of the risk to pregnant women at ANC. Data from this study shows that 66% of participants received three or more IPTp-SP doses. This finding is also consistent with previous investigations in other parts of Ghana reporting that more than 60% of pregnant women took at least three IPTp-SP doses [5, 27]. It is interesting to note that the fairly satisfactory coverage of IPTp-SP in Ghana does not seem to differ significantly among community settings across the country. The study shows that a small number of women (N = 14) nevertheless reported not taking IPTp-SP during pregnancy due to not attending ANC. This confirms the importance of ANC attendance in IPTp-SP coverage, especially since it is administered in Ghana only as a direct observation therapy (DOT). It appears, therefore, legitimate to further consider the involvement of communities in the delivery of IPTp-SP to all eligible pregnant women, as this would help reduce the number of women not taking IPTp-SP due to their non-attendance at an ANC.

In this study, a beneficial effect of IPTp-SP on birth weight was observed. In this instance, uptake of ≥ 3 SP doses was clearly associated with an increase in birth weight at the two study sites by 360 g (Table 3). The association of more IPTp-SP doses with increased birthweight was obvious in both primigravid and multigravid women and consistent with a study by Desai et al. [11] and Quakyi et al. [5] that reported reduced risk of LBW among pregnant women living in medium–high SP resistant areas. In this study, no association between IPTp-SP dosage and parasite density or placental infection at delivery was observed, as in other studies [6, 28]. As discussed previously by Quakyi et al. [5], the lack of an observable link in this non-longitudinal study is not surprising and does not necessarily reflect any lack of effect on parasites. It’s still plausible that the effect of SP treatment on other non-malarial infections (especially sexually transmitted infections) indirectly contributes to this observed beneficial effect on birth weight. Also, an association was not found between uptake of ≥ 3 SP doses and anaemia confirming the finding of Agyeman et al. [29]: IPTp-SP alone doesn’t protect pregnant women from anaemia, because anaemia in pregnancy is multifactorial and involves other factors such as nutrition.

Further, the study shows a high prevalence of *Pfdhfr*/*Pfdhps* quintuple mutations in parasite isolates from both study sites, but unlike what is happening in East Africa, no *Pfdhps* K540E mutation was found [30]. This result indicates that *Pfdhps* K540E mutation conferring a high level of resistance to SP has not yet emerged at the study areas, although it has been detected at very low frequency at different communities in southern Ghana [15].

Between first ANC and delivery, the prevalence of the A581G *Pfdphs* mutation increased approximately 3.9-fold in the study population, suggesting that IPTp-SP use may possibly select the *Pfdhps* A581G mutation. This observation is further supported by the fact that the prevalence of *Pfdphs* A581G mutation increased with the number of SP doses taken, as also described by Tornyigah et al. [15]. The correlation of this mutation with IPTp-SP treatment is consistent with selection due to drug pressure. It has been shown that *Pfdhps* A581G mutation occurring on a *Pfdhfr/Pfdhps* quintuple mutation background was associated with higher risks of infection in Malawian pregnant women [31]. Also, no correlation was found between parasite density and the frequency of the *Pfdhps* A581G mutation at the study sites. However, the increase in the prevalence of *Pfdhps* A581G mutation at delivery and the emergence of the sextuple mutation (**IRN**I-**A/FG**K**GS/T)** raises the question of whether a possible increase in the prevalence of parasites carrying the sextuple mutation could gradually lead to IPTp-SP drug failure in Ghana.

The results obtained during this study showed that parasite SP resistance background did not affect birth weight outcomes as IPTp-SP was associated with an increase in birth weight in the study sites but also as shown in different communities in southern Ghana irrespective of SP resistance [5]. This finding may be due to secondary non-malaria related effects (bacterial or fungal genital infections for instance), that also impact on fetal growth and maternal health [32]. Therefore, it would be interesting to examine the effect of SP on birth weight independent of malaria infection.

Despite the high prevalence of *Pfdhfr/Pfdhps* quintuple mutations and even the existence of sextuple mutations, IPTp-SP continues to prove its beneficial effect on birth weight at delivery in Ghanaian pregnant women.

## Study limitations

Although this study made it possible to highlight some important associations, it nevertheless has some limitations which need to be pointed out. One of them has been the inability of the team to have sufficient microscopy data at the time of delivery to be able to study the part of associations attributable to microscopic and sub-microscopic infections.

Although there was an attempted to control known confounders in the design and at the analysis stage, it is possible that the use of ITNs among participants that have not been included in the analysis could have influenced the various observations made.

The fact that this study was observational and did not involve any intervention allowed to have a real picture of what is happening on the field, but remains a weakness because they only offer one point of observation.

## Conclusions

The NMCP target is to treat all pregnant women with IPTp-SP even though this will undoubtedly lead to a drastic increase of SP-induced drug pressure on parasite populations. Thus, an increase in A581G mutation prevalence and/or emergence of the K540E mutation must be carefully documented to assure long-term IPTp-SP efficacy and performed in parallel with implementation of field studies on alternatives to IPTp-SP such as other anti-malarial drug combinations or a malaria vaccine for pregnant women.

## Data Availability

The datasets analysed for this study are included in the article, additional data required are available from the corresponding author on reasonable request.

## Abbreviations

ANC: Antenatal care visit or Antenatal care consultation
CHIPS: Community-based Health Planning and Services
DOT: Direct observation therapy
EIR: Entomological inoculation rate
Hb: Haemoglobin
IPTp: Intermittent preventive treatment of malaria in pregnancy
LBW: Low birth weight
MiP: Malaria in pregnancy
NMCP: National Malaria Control Programme
NMIMR: Noguchi Memorial Institute for Medical Research
RDT: Rapid diagnostic test
SP: Sulfadoxine-pyrimethamine
WHO: World Health Organization
